# Green light irradiation during sex differentiation induces female-to-male sex reversal in the medaka *Oryzias latipes*

**DOI:** 10.1038/s41598-019-38908-w

**Published:** 2019-02-20

**Authors:** Oki Hayasaka, Yutaka Takeuchi, Kazuhiro Shiozaki, Kazuhiko Anraku, Tomonari Kotani

**Affiliations:** 1 The United Graduate School of Agricultural Sciences, Kagoshima University, Kagoshima, 890-0056 Japan; 2 Faculty of Fisheries, Kagoshima University, Kagoshima, 890-0056 Japan

## Abstract

This study investigated whether irradiation of a specific light wavelength could affect the sex differentiation of fish. We first found that the photoreceptor genes responsible for receiving red, green, and ultraviolet light were expressed in the eyes of medaka during the sex differentiation period. Second, we revealed that testes developed in 15.9% of genotypic females reared under green light irradiation. These female-to-male sex-reversed fish (i.e. neo-males) showed male-specific secondary sexual characteristics and produced motile sperm. Finally, progeny tests using the sperm of neo-males (XX) and eggs of normal females (XX) revealed that all F1 offspring were female, indicating for the first time in animals that irradiation with light of a specific wavelength can trigger sex reversal.

## Introduction

Light is one of the important environmental factors for aquatic habitats. Whereas the physiological mechanism of the effects of light, i.e. photoperiod and intensity, in growth and reproduction has been studied in many animal taxa^1,2^, the knowledge on the effects of the wavelength, i.e. the color of lights, to physiological states of animals is limited. For example, in teleost fish, irradiation by green light stimulates somatic growth by upregulating melanin-concentrating hormone (MCH)^3,4^. In addition to enhanced growth performance, positive effects on feeding behavior and stress relief have been reported in several fish species by irradiation with specific light wavelength^5–9^.

In some fish species, environmental factors such as water temperature^10,11^, pH, rearing density and photoperiod^12^ affect genotype-dependent sex differentiation and induce sex-reversal^13,14^. The mechanism of masculinization by high water temperatures has been well studied. In juveniles of the Japanese flounder (*Paralichthys olivaceu*s) exposed to high water temperatures, overproduced cortisol, a stress marker in fish, directly suppresses the expression of the *cyp19a1* gene, which codes for an estrogenic enzyme that converts androgen to estrogen and subsequently induces masculinization^15^. In addition to this stress-induced sex-reversal mechanism, it has recently been reported that masculinization caused by exposure to high water temperatures in the European sea bass (*Dicentrarchus labrax*) results from an increase in the *cyp19a* promoter methylation level in females, indicating that water temperature-induced masculinization involves DNA methylation-mediated control of aromatase gene expression^16^. Although the fundamental mechanism of environmental sex determination (ESD) by temperature has been studied at the molecular levels, nothing is known about the effects of specific light wavelengths on gonadal development and sex differentiation.

Medaka (*Oryzias latipes*) is a small model fish with several desirable features, including a short generation time, small genome size, and the availability of an inbred strain, Hd-rRII1, showing genotypic sex-dependent body color^17,18^. Their sex determination system is male heterogametic (XY/XX). A sex determining gene named *dmy*/*dmrt1bY* (*DM-domain gene on Y chromosome*) has been identified on the Y chromosome^19–21^. In the Hd-rRII1 strain, body color is different in males and females. The allele *R* of the *r* locus (a sex-linked pigment gene) is located on the Y chromosome. In this strain, X^*r*^X^*r*^ females have a white body color, whereas X^*r*^Y^*R*^ males have an orange-red body color^21^. Therefore, it is easy to detect sex-reversed fish by observation of phenotypic sex (i.e. sex of gonads and secondary sexual characteristics appearing on fins) and determination of genotypic sex (i.e. genomic PCR for the *dmy* gene and body color). In addition, medaka normally follow a strong genetic sex determination system that is not easily influenced by environmental factors^22^. However, the induction of sex-reversal by administration of sex-steroid hormone, exposure to high water temperature, and the regulation of primordial germ cell number has been demonstrated^10,15,23–27^. These reports suggest that artificial sex control is possible in this species. Therefore, we selected medaka for molecular genotypic analysis of sex-reversal induced by irradiation with specific light wavelengths.

Rod and cone photoreceptor cells of the retina enable the detection of light and the initiation of visual signaling^17^. Cone photoreceptors become functional under the sufficient light condition and they are related to the color vision. The spectral range of detection is controlled by the selected expression of visual pigments differing in absorption spectra in cone photoreceptor cells. Cone opsins, which belong to the subfamily of G-protein-coupled transmembrane receptors and form wavelength-specific visual pigments together with retinal chromophores, play key roles in color discrimination and in the appropriate processing of post-receptor signals^17,18,28^. Fish possess many cone opsin gene orthologs responsible for photoreception, which have emerged though gene duplication and allow fishes to adapt to the various light conditions of the aquatic environment^29^. In the retinas of adult medaka, 8 cone opsin genes, such as ultraviolet (*SWS1*), blue (*SWS2-A*, *SWS2-B*), green (*RH2-A*, *RH2-B*, *RH2-C*), and red (*LWS-A* and *LWS-B*) are responsible for photoreception with retinal chromophores and have already been identified^30^. However, it has not been revealed which opsin genes are expressed in the eyes of medaka during the sex differentiation stage.

In the present study, we first investigated opsin gene expression in the eyes of 3 day after hatched medaka embryos. To test whether irradiation of a specific wavelength could affect sex differentiation in medaka, newly hatched medaka embryos were reared under white and green light-emitting diodes (LED) for 3 months. The male-to-female and female-to-male sex reversal rates were studied by comparing the phenotypic sex with the genotypic sex. To confirm whether the sex-reversal was functional or not, a progeny test was conducted by artificial insemination, and the development and sex of F1 offspring were analyzed.

## Results

### Green opsin genes were expressed in eyes of newly hatched Hd-rRII1 medaka

**Figure 1:**
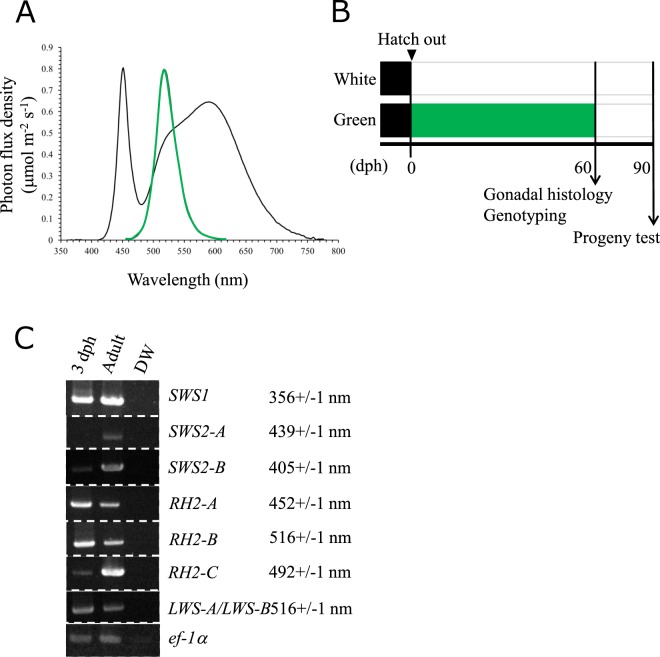
Rearing conditions and opsin gene expression of newly hatched medaka. (**A**) White LED shows two peaks at 451 and 581 nm. Green LED shows one peak at 518 nm. (**B**) Experimental schedule. White and green LED irradiations started from just after hatching (0 dph) to 60 dph. Fish were sampled at 60 dph for gonadal histology and genotyping. Progeny tests were conducted at 90 dph by artificial insemination. (**C**) RT-PCR analysis of 8 opsin genes and *ef-1a* in the eyes of 3 dph and adult medaka. The λ_max_ values for each opsin protein are from Matsumoto *et al*. 2006^30^.

All 8 opsin genes were expressed in the eyes at the adult stage, whereas only 5 opsin genes were expressed in the eyes at the 3 day after hatch (Fig. 1C). The ultraviolet opsin gene (*SWS1*), all three types of green opsin genes (*RH2-A*, *RH2-B*, *RH2-C*), and two type of two red opsin genes (*LWS-A/LWS-B*) were expressed in the eyes of 3 day after hatched medaka (Fig. 1C). In contrast, two types of blue opsin genes (*SWS2-A* and *SWS2-B*) were not expressed at the 3 day after hatch medaka (Fig. 1C).

### Appearance of genotypic females showing male-specific phenotypes

**Figure 2:**
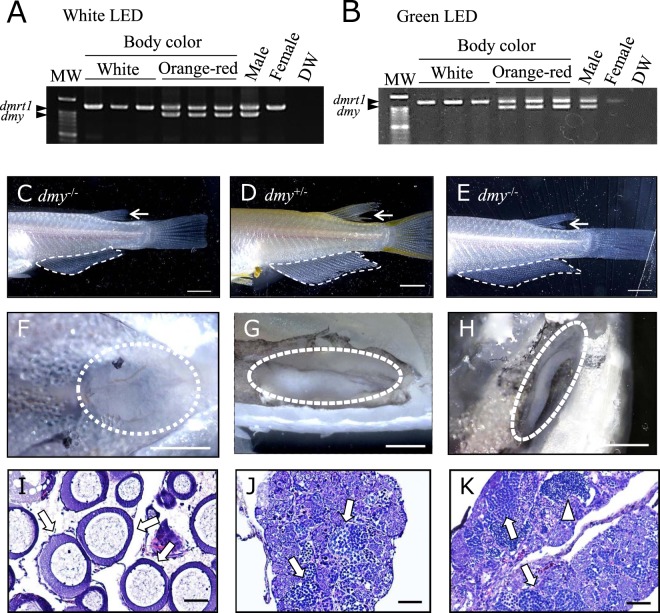
Determination of genotypic and phenotypic sex by body color, genomic DNA PCR, and the secondary sexual characteristics of dorsal and anal fins. (**A**,**B**) Genotypes of white and orange-red body color of 60-dph Hd-rRII1 medaka reared under white LED (**A**) and green LED (**B**). A primer set designed for the conserved region of dmy, a sex determination gene on the Y chromosome, and dmrt1, an orthologue of dmy located on an autosome, were used for PCR analysis. Fin clips of adult males and females were used as positive controls. DW; distilled water for negative control. MW; molecular weight marker. (**C**) Genotypic female (white body color) showing female-specific fin types under green LED irradiation. An uncut dorsal fin (arrow) and a round-shaped anal fin (dotted line) are secondary sexual characteristics of females. (**D**) Genotypic male (orange-red body color) showing male-specific fin types under green LED irradiation. A deeply cut dorsal fin (arrow) and a parallelogram-shaped anal fin (dotted line) are secondary characteristics of males. (**E**) Genotypic female (white body color) showing secondary characteristics of males, i.e. a deeply cut dorsal fin (arrow) and a parallelogram-shaped anal fin (dotted line). (**F**–**H**) External observation of the gonads of green LED-irradiated medaka (**C**–**E**). Dotted lines show ovary (**F**) and testes (**G**,**H**). (**I**–**K**) Histological observation of gonads of green LED-treated medaka (**C**–**E**). Perinucleolar oocytes (arrows in I) in ovaries and spermatogenic cells (arrows in **J**) were observed. Spermatogenic cells (arrows) including spermatozoa (arrowheads) were observed in female-to-male sex-reversed medaka testes (**K**). Scale bars = 2 mm (**C**–**H**), 40 μm (**I**–**K**).

The experimental fish showed a high survival rate in each LED irradiation treatment (over 80%) at 60 dph. In both white and green LED treatment groups, *dmy* and *dmrt1* genes were detected in all orange-red body colored individuals, while only the *dmrt1* gene was detected in white body colored individuals by genomic DNA PCR (Fig. 2A,B), indicating that the body color represented the genotypic sex even if the fish were reared under green LED irradiation.

In the green LED treatment group, all genotypic males (*dmy*^+/−^, orange-red body color) had spermatogenic testes and male-specific anal and dorsal fins (n = 40) (Fig. 2D,G,J, Table 1). Notably, the fish showed male-specific secondary sex characteristics, i.e. parallelogram-shaped anal fins and deeply cut dorsal fins, which were observed in 15.9% of genotypic females (*dmy*^−/−^, white body color, n = 7) (Fig. 2E). External and histological observation revealed that those fish possessed testes, spermatogenic germ cells and mature spermatozoa (Fig. 2H,K) (Supplementary Fig. 4). The other 84.1% of genotypic females (n = 37) possessed female-specific fin types, ovaries, and oocytes (Fig. 2C,F,I, Table 1). The appearance rate of sex-reversed fish under green LED treatment was significantly higher (*p* < 0.05) than under white LED treatment (Table 1). No sex-reversed fish was observed in the white LED irradiation group (n = 38, Table 1).

**Table 1:** Sex reversal rates (%) obtained by irradiation with white and green LEDs.

LED irradiation	Genotype (*dmy*)	−/−	−/−	+/−	+/−
	Phenotype	Ov	Tes	Ov	Tes
White		100	0^a^	0	100
		(18)	(0)	(0)	(20)
Green		84.4	15.9^b^	0	100
		(37)	(7)	(0)	(40)

Each alphabetical (a, b) subscript indicates the result of a Fisher’s exact test among treatments (*p* < 0.05) (a < b). Numbers of fish observed are shown in parentheses.

### Sex-reversed males (XX) produced functional spermatozoa and all-female F1 offspring

**Figure 3:**
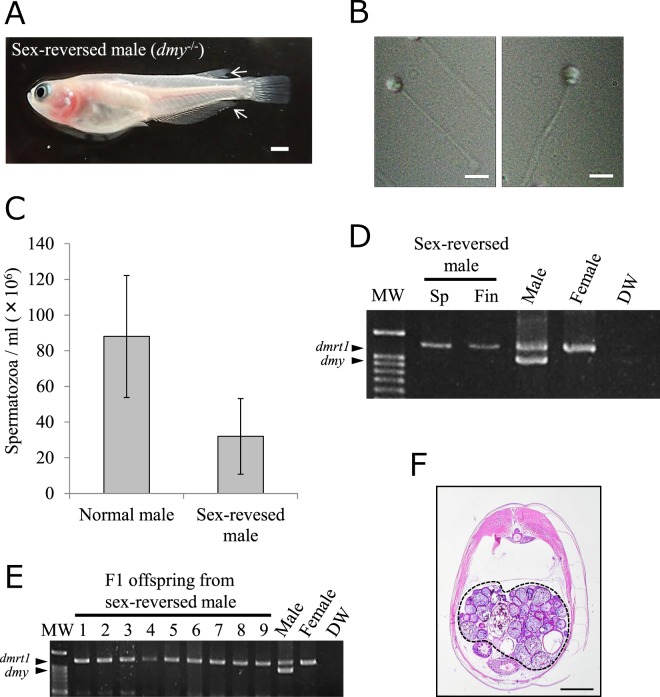
Progeny test of sperm obtained from sex-reversed males. (**A**) A sex-reversed male showing a female genotype (*dmy*^−/−^), female-specific white body color, and male-specific secondary sexual characteristics in fins (a parallelogram dorsal fin and a deeply cut anal fin, arrows). (**B**) Spermatozoa obtained from a sex-reversed male (left) were morphologically normal and indistinguishable from those of a normal male (right). (**C**) Density of spermatozoa of normal males (n = 3) and a sex-reversed male (n = 2). *P* < 0.05. (**D**) Genomic DNA PCR of the sperm (Sp) and a fin (Fin) of a sex-reversed male using a primer set for *dmy*/*dmrt1* genes. (**E**) Genomic DNA PCR for F1 offspring obtained from sex-reversed males (n = 2) (lanes 1–9) using a primer set for *dmy/dmrt1* genes. Fin clips of adult males and females were used for positive controls. DW, distilled water for negative control; MW, molecular weight marker. (**F**) Histological observation of a gonad of F1 offspring. Dotted line shows an ovary. Scale bars = 2 mm (**A**), 10 mm (**B**), 0.5 mm (**F**).

Motile sperm was obtained from sex-reversed males (white body color, *dmy*^−/−^, male-specific fin types) (n = 2, Fig. 3A) (Supplementary Movie 1). The spermatozoa were morphologically indistinguishable from those of normal males (Fig. 3B). The density of spermatozoa of the sex-reversed males was approximately 50% lower than that of normal males (Fig. 3C). Genomic DNA PCR analysis revealed that the *dmy* gene was detected neither in sperm nor in the fins of sex-reversed males (Fig. 3D).

To test whether sex-reversed males could show sexual behavior, each male (n = 2) was paired with normal females (n = 7) and reared in optimal spawning conditions for one month. However, no sexual behavior and spawning was observed. Therefore, we conducted artificial insemination using sperm stripped from sex-reversed males (*dmy*^−/−^) and eggs from normal females (*dmy*^−/−^). Genomic PCR analysis revealed that none of the F1 offspring analyzed (n = 18) possessed *dmy* (Fig. 3E). Gonadal histology revealed that all of the F1 offspring (n = 18) at 2-months-old possessed ovaries (Fig. 3F).

## Discussion

In this study, we found that irradiation with green LED light during the sex differentiation period induced female-to-male sex-reversal production of fertile sperm in genotypic female, indicating for the first time in animals that irradiation of a specific wavelength can be a trigger for sex reversal.

In the wild, the frequency of female-to-male sex-reversal in medaka is 0.98% in Japan (Northern and Southern populations), China, and West and East Korea^31^. The female-to-male sex-reversal rate of the Hd-rRII1 strain (15.9%) obtained in this study was much higher than that observed in wild populations. Spontaneous XX male were also reported in inbred strain medaka^32^. However, spontaneous XX male was not observed in this experiment. In addition, female-to-male sex-reversal induced by green LED irradiation was not observed in the commercially available orange-red variety (data not shown). It has been reported that, in high water temperature treatment, the female-to-male sex-reversal rate varies among strains (e.g. 50% in the HNI strain and 24% in the Hd-rRII1 strain)^10^. Therefore, differences of genetic and epigenetic background could influence sex reversal rates resulting from green LED irradiation. Further studies are needed to test whether UV and red light can affect gonadal differentiation, because the opsin genes responsible for photoreception of these wavelengths (*SWS1* and *LWS-A*) were expressed in the eyes of 3 day after hatch medaka. Other factors of larval rearing conditions, such as the irradiation period and light intensity, need to be investigated to reveal the requirements for successful sex-reversal and obtain higher sex-reversal rates.

In this study, irradiation of green LED light was tested based on the opsin gene expression profile in the eyes of 3 day after hatched medaka. However, photoreceptors exist not only in the retina, but also in the pineal gland and deep brain of medaka^33^. In *Oncorhynchus masou*, the saccus vasculosus, which is located in the deep brain, is known to express the rhodopsin family genes *RH1*, *SWS1*, *LWS*, and *OPN4* and regulate photoperiod gonadal development^2^. Interestingly, in the jellyfish *Clytia gregaria*, it was revealed that the gonadal photoreceptor known as opsin 9 regulates gonadal development^34^. Thus, it could be that known and unknown photoreceptive organs influence sex differentiation in fish^35^. An important issue raised by this study is which photoreceptive organ that received the green light induced the female-to-male sex-reversal in medaka.

The mechanism by which female-to-male sex reversal occurred via green light in medaka was not revealed in this study. According to our results, two hypotheses were proposed. In medaka, sex reversal can be induced by exposure to high water temperature^10,15,23,24,35^. The expression of the *gr* gene encoding a glucocorticoid receptor, a stress marker, is increased by exposure to high water temperature during the sex differentiation period^15^. Thus, it has been proposed that overproduction of glucocorticoid suppresses *cyp19a*, a key aromatase gene that converts androgen to estrogen and induces female-to-male sex-reversal^15,35^. One possibility is that the stress caused by a specific light wavelength induced elevated cortisol levels and consequently suppressed aromatase gene expression in our study. Another possibility is that the effect of cell death induced sex reversal via irradiation of a specific wavelength. In insects, the common fruit fly *Drosophila melanogaster*, house mosquito *Culex pipiens molestus*, confused flour beetle *Tribolium confusum*, and strawberry leaf beetle *Galerucella grisescens* are killed by irradiation with visible blue light^36,37^. These studies suggest that irradiation with blue light produces reactive oxygen species (ROS) and induces cell death. In normal gonadal development of medaka, genotypic females possess 5 times more germ cells at 10 dph than genotypic males^23^. The depletion of germ-cell numbers by RNA knockdown can induce female-to-male sex-reversal^26,27^, suggesting that the number of germ cells in embryonic gonads is a factor for phenotypic sex differentiation of medaka. Therefore, if excessive irradiation with a specific wavelength of light can produce ROS, it might suppress germ cell division and induce female-to-male sex-reversal.

In many species of cultured finfish, females exhibit higher growth rates than males and attain larger sizes, because the males are often sexually mature before reaching marketable size and show retarded growth rates. For example, *Paralichthys olivaceus* and *Nibea albiflora* females grow faster than males^38,39^. In the sturgeon family, their eggs (i.e. caviar) have high value in the market^40,41^. Therefore, sex manipulation of fish and monosex culture of females is desirable for commercial operations^42–44^. In the case of male heterogametic species, gynogenesis followed by androgenic hormonal sex reversal of females into males theoretically allows for the production of 100% sex-reversed XX males^45,46^. Conventional techniques for practical sex-reversal are the treatment of sexually undifferentiated fry by offering feed treated with or immersion with hormones or hormone analogues, which has been shown to work well under carefully controlled conditions in a wide range of species^47^. However, excessive doses of some hormones can lead to sterility or abnormal gonadal development. In addition, concerns persist over the safety of commercial sex reversal treatments both with regard to the safety of the farmer and of the consumer together with possible environmental impacts. If irradiation with a specific wavelength of light could induce neo-males in other aquaculture target species, we can expect the establishment of a new sex manipulation system that is safer and less costly than using sex hormones.

## Materials and Methods

### Animals and ethics

The HdrR-II1 (Strain ID: IB178) inbred strain of medaka, supplied by NBRP Medaka (https://shigen.nig.ac.jp/medaka/), was used for all experiments. The animal experiments in this study were approved by the Kagoshima university animal experiment committee and all experiments were performed in accordance with the guidelines for the care and use of laboratory animals of Kagoshima university.

### Opsin gene expression of juvenile medaka

Eight cone opsin genes, *LWS-A*/*LWS-B*, *RH2-A*, *RH2-B*, *RH2-C*, *SWS1*, *SWS2-A*, and *SWS2-B*, have been reported in medaka^30^. The expression of opsin genes in the eyes of newly hatched embryo medaka (3 days post-hatching, dph) of the Hd-rRII1 inbred strain was analyzed by RT-PCR. Each opsin gene primer was from Matsumoto *et al*.^30^ and Chinen *et al*.^48^. *LWS-A* and *LWS-B* genes were highly similar coding^30,49^. Therefore, we used only *LWS-A* primer set. The total RNA was extracted from the eyes after homogenization in a 1.5-ml tube with 1000-μl Tri Reagent (Cosmo Bio Co., Ltd. Japan) following the manufacturer’s instructions. A ReverTra Ace kit (ReverTra Ace qPCR RT Master Mix with gDNA Remover; Toyobo Co., Ltd. Japan) was used for cDNA synthesis, and BIOTAQ DNA polymerase (Bioline Ltd. United Kingdom) was used for RT-PCR. PCR conditions were as follows: 95 °C for 30 sec, then 40 cycles of 95 °C for 30 sec, 55 °C for 30 sec, 72 °C for 1 min, and 80 °C for 8 sec.

### Rearing condition of medaka

The fish were reared under each color of LED (LDA6-G and LB1526G, Beamtec Co., Ltd. Saitama, Japan) in rectangular parallelepiped glass tanks (31.5 × 18.5 × 24.4 cm) covered by shielding curtains. The distance from the water surface to the light was approximately 5 cm. The light wavelength peaks were predetermined by an illuminance spectrophotometer (CL-500A; Konica Minolta, Inc., Tokyo, Japan) and were 450 nm for the white LED and 518 nm for the green LED (Fig. 1A). The photoperiod in the rearing term was controlled at 14-h light and 10-h dark, and water temperate was maintained at 26 ± 5 °C. The formula food (Kyorin Co., LTD, Hyogo, Japan) and *Artemia* nauplii hatched from commercialized eggs (Brine Shrimp EGGS-90, Kitamura co., ltd, Kyoto, Japan) were fed to fish from newly hatched to adult. Each LED was used to irradiate fish tanks from 0 to 60 dph. Progeny tests were performed at 90 dph (Fig. 1B).

### Determination of genotypic sex of medaka

The genotypic sex of medaka was determined by genomic DNA PCR for the *dmy* gene and the observation of body color. A 25-mm^2^ sample of caudal fin was dissolved in 500 μl of cell lysis regent (100 mM Tris-Cl, 5 mM EDTA, 200 mM NaCl, 0.2% SDS, 1 μl Proteinase K) at 55 °C for 12 h in an incubator. After 10 min of centrifugation at 13,000 × *g*, the supernatant was mixed with 500 μl of isopropanol, and the precipitant was washed with 70% alcohol. The genomic DNA was eluted with TE buffer. The genomic DNA PCR primers were from Matsuda *et al*.^20^. One primer set can detect two genes that are *dmy* (1000 bp) and *dmrt1* (1400 bp). Because *dmy* only exists in males and *dmrt1* exists in both sexes, genotypic males show two bands and genotypic females show one band by genomic DNA PCR. BIOTAQ DNA polymerase was used for genomic DNA PCR. PCR conditions were as follows: 96 °C for preheating and 94 °C for 2 min, then 35 cycles of 94 °C for 1 min, 55 °C for 1 min, 72 °C for 1 min, and 72 °C for 7 min. In the Hd-rRII1 strain, genotypic males and females show orange-red and white body color, respectively. Therefore, body color was used to discriminate the genotypic sex of this inbred strain. As an internal control, the *ef-1*α primer set was used according to Nakamoto *et al*.^50^.

### Determination of phenotypic sex of medaka

The phenotypic sex was determined with gonadal histology and observation of shapes of dorsal and anal fins. The shapes of the anal and dorsal fins represent the secondary sex characteristics of medaka^51^. Phenotypic females show round and short anal fins (Fig. 2C), but phenotypic males show sharp and long anal fins (Fig. 2D). In addition, the dorsal fins of phenotypic males are deeply cut (inset of Fig. 2D), but those of phenotypic females are uncut (inset of Fig. 2C).

The gonads were fixed in Bouin’s fixatives at 4 °C in a refrigerator. The fixed gonads were dehydrated in a graded series of ethanol from 70% to 100% and cleared by xylene. In all processes, a shaker (Wave-SI; Taitec Co., Japan) was used. The gonads were embedded in paraffin and sectioned from 4 to 6 μm by a microtome (HM 315 S; MICROM international GmbH, Ltd. Germany). The sections were stained by hematoxylin and eosin (H&E). The sections were deparaffinized for 5 min in xylene and dehydrated in a graded series of ethanol from 99% to 70%. The sections stained with hematoxylin for 2 min and eosin for 10 min were washed in tap water. Finally, the sections were stained with eosin and dehydrated in a graded series of ethanol from 70% to 99% and mounted by cover glass with encapsulating material (Entellan New; Merck & Co., USA).

### Counting of spermatozoa and progeny tests by artificial insemination

The sperm was obtained from anesthetized sex-revered males and normal males. The spermatozoa were counted three times in each fish by a hemocytometer (Burker-Turk; Sigma-Aldrich Co. LLC, USA).

For artificial insemination, mature sex-reversed fish (HdrR-II1) and intact females (orange-red variety) were separately reared in glass tanks in spawning conditions as described above. The sperm were obtained from sex-reversed fish with abdominal pressure. The unfertilized eggs were obtained from anesthetized females. Those eggs and spermatozoa were mixed in wells of 6-well culture plates with artificial Iwamatsu’s seminal plasma (pH 7.3)^52^. The fertilized eggs were reared at 26 °C, and the obtained newly hatched embryos were reared for two months. The genotypic and phenotypic sex of the F1 offspring were determined as described above.

### Statistics

Fisher’s exact test was used for the appearance rate of sex-reversed fish and the sex ratio of F1 offspring. T-tests were used for spermatozoa counts. All data were analyzed using the statistical analysis software Stat View 5.0) SAS Institute Inc. NC, USA).

## Supplementary information

Supplementary Figure 1. Supplementary Figure 2. Supplementary Figure 3. Supplementary Figure 4.

Supplementary Movie 1.
