# Supplementary material for: Green light irradiation during sex differentiation induces female-to-male sex reversal in the medaka Oryzias latipes
Source: Sci Rep. 2019 Feb 20;9:2383. doi: 10.1038/s41598-019-38908-w (PMC6382872; doi:10.1038/s41598-019-38908-w)
Supplement: Supplementary file 1 — Supplementary Figure 1. Supplementary Figure 2. Supplementary Figure 3. Supplementary Figure 4. [file 41598_2019_38908_MOESM1_ESM.pdf]

Green light irradiation during sex differentiation induces female-to-male sex reversal in the medaka *Oryzias latipes*

Oki Hayasaka[1], Yutaka Takeuchi[1,2], Kazuhiro Shiozaki[1,2], Kazuhiko Anraku[1,2], Tomonari Kotani[1,2*]

[1] The United Graduate School of Agricultural Sciences, Kagoshima University, Kagoshima 890-0056, Japan
[2] Faculty of Fisheries, Kagoshima University, Kagoshima 890-0056, Japan

Corresponding: Tomonari Kotani, Faculty of Fisheries, Kagoshima University, Shimoarata 4-50-20, Kagoshima 890-0056, Japan.
Email: kotani@fish.kagoshima-u.ac.jp

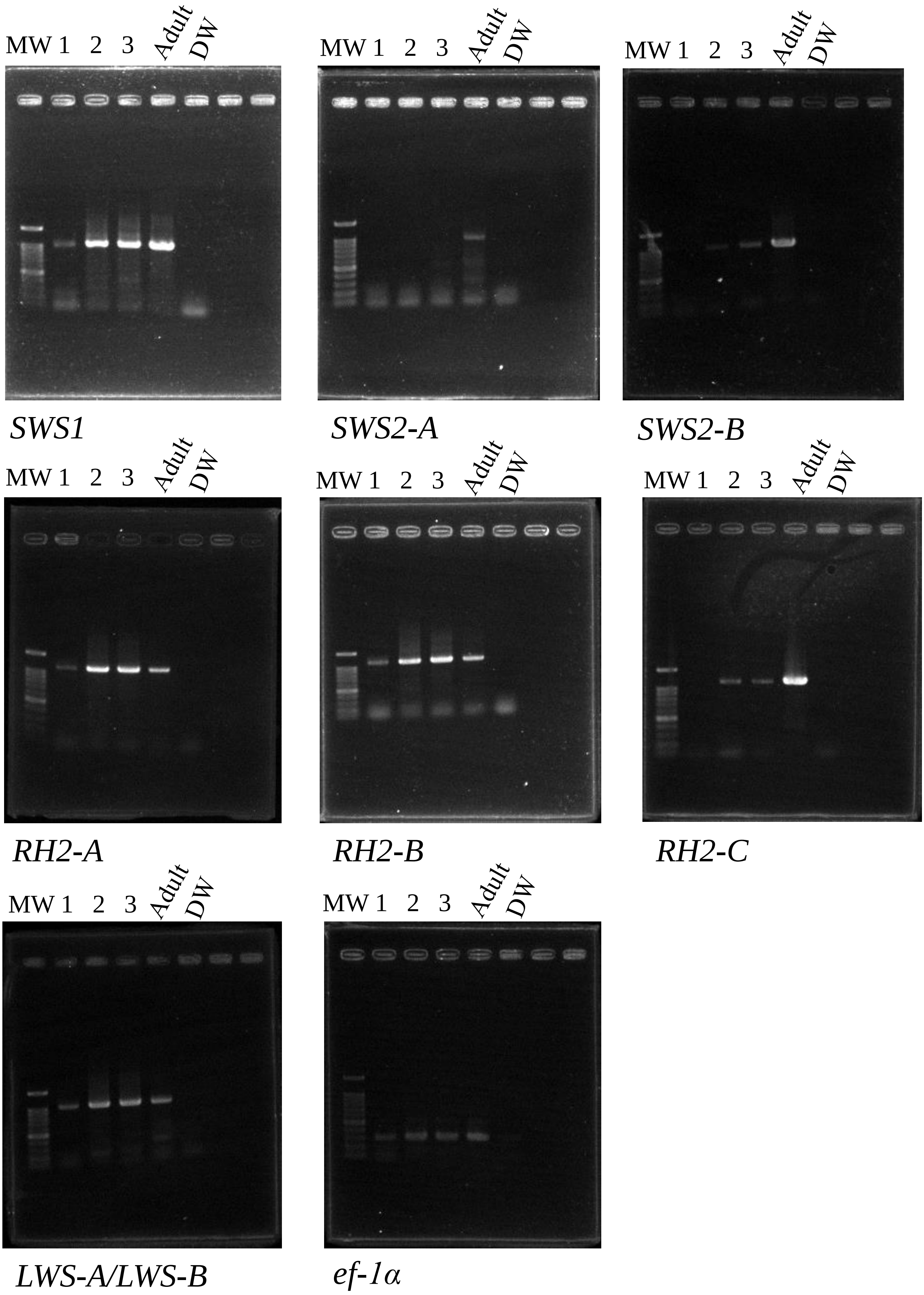

Supplementary Figure. 1.

RT-PCR analysis of 8 opsin genes and *ef-1α* in the eyes of 3 dph and adult medaka.

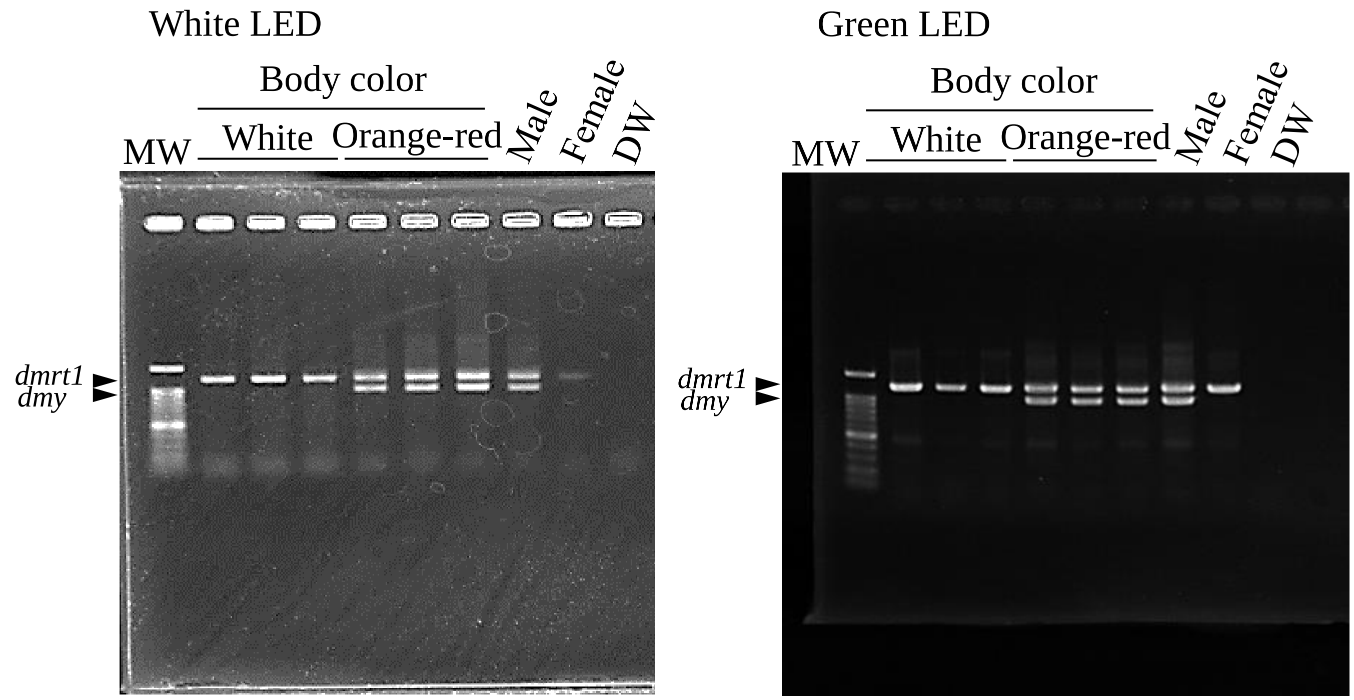

A B

Supplementary Figure. 2.

A and B) Genotypes of white and orange-red body color of 60-dph Hd-rRII1 medaka reared under white LED (A) and green LED (B)

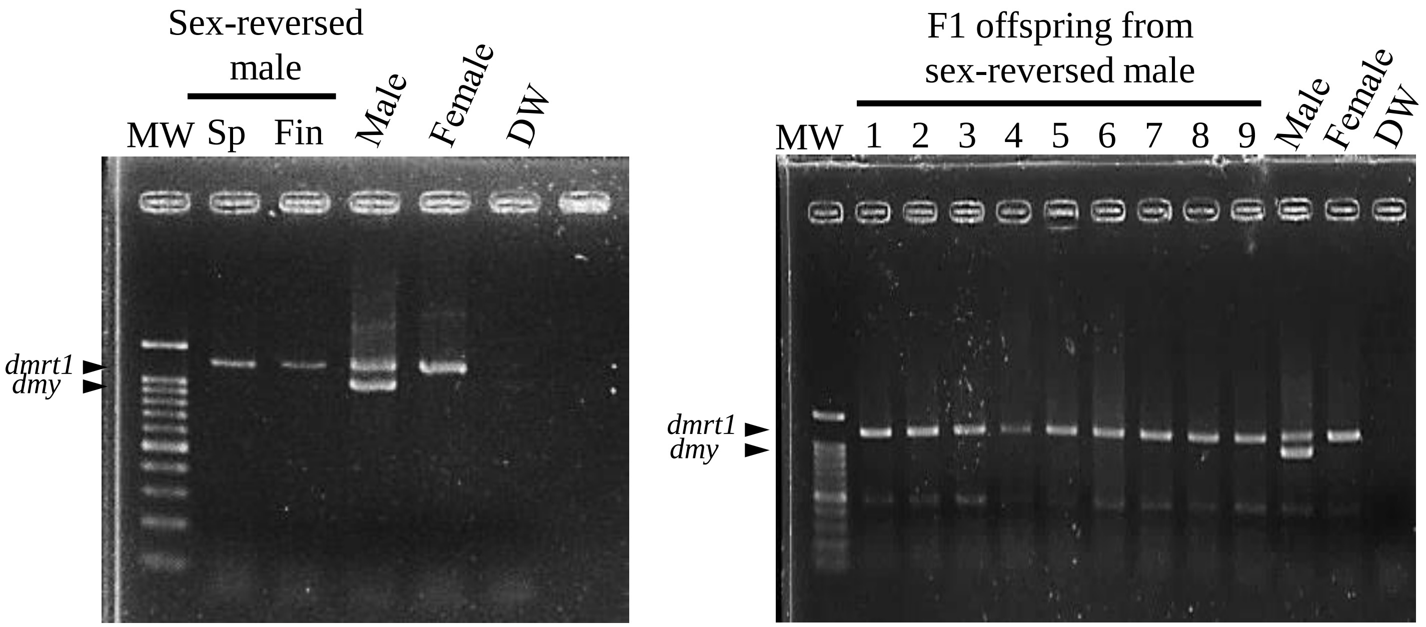

A B

Supplementary Figure. 3.

A) Genomic DNA PCR of the sperm (Sp) and a fin (Fin) of a sex-reversed male using a primer set for *dmy*/*dmrt1* genes.

B) Genomic DNA PCR for F1 offspring obtained from sex-reversed males (lanes 1-9) using a primer set for *dmy*/*dmrt1* genes.

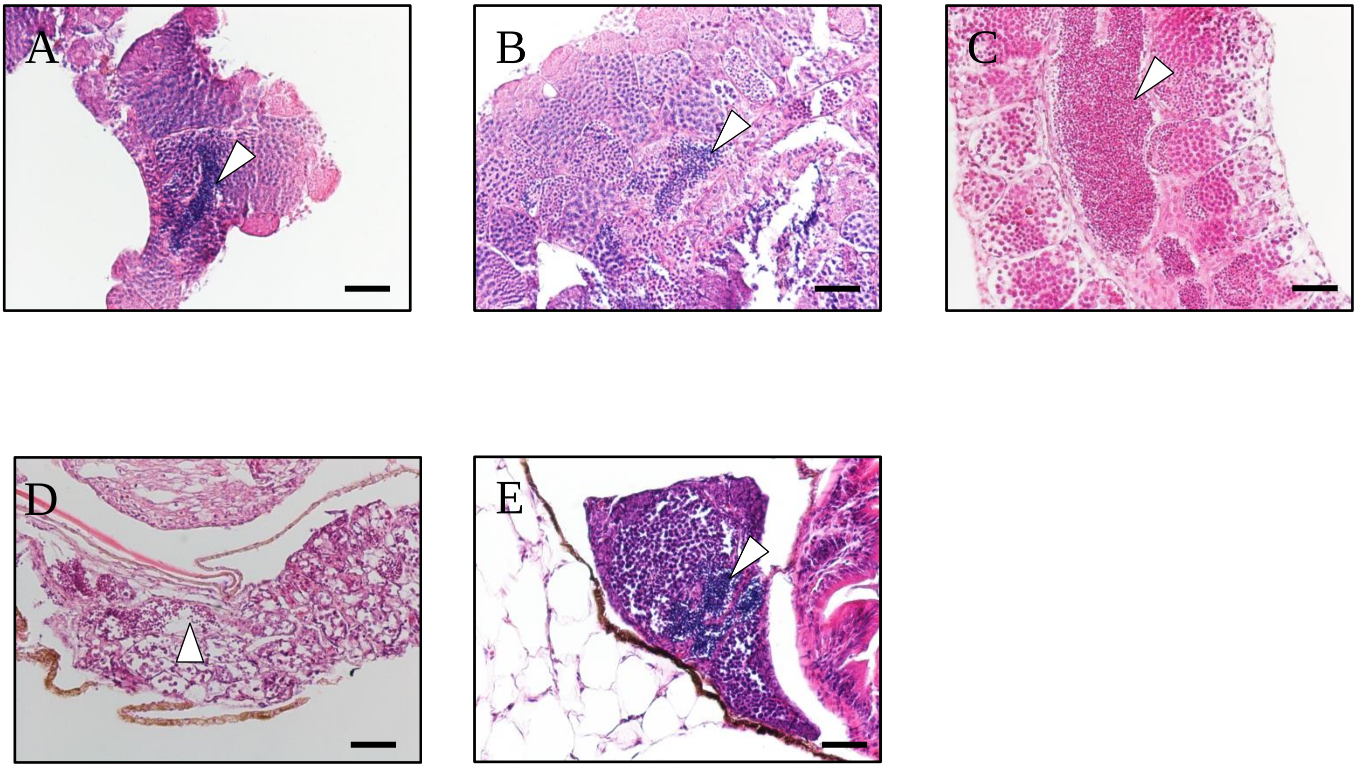

Supplementary Figure 4.

Histological observation of gonads of neomales. Spermatogenic cells including spermatozoa (arrowheads) were observed in female-to-male sex-reversed medaka testes. Scale bars = 40 μm (A, B, C, D, E).
